# The Impact of an Acute Active Reading Intervention on Physical Activity Levels in Preschoolers: A Comparative Analysis

**DOI:** 10.3390/children11020183

**Published:** 2024-02-02

**Authors:** Danielle D. Wadsworth, Katherine E. Spring

**Affiliations:** 1 Exercise Adherence and Obesity Prevention Laboratory, School of Kinesiology, Auburn University, Auburn, AL 36849, USA; katie.spring@pbrc.edu; 2 Pediatric Obesity and Health Behavior Laboratory, Division of Population and Public Health Science, Pennington Biomedical Research Center, Baton Rouge, LA 70808, USA

**Keywords:** active play, moderate-to-vigorous physical activity, fundamental motor skill competence

## Abstract

The purpose of this study was to examine the acute effects of an active reading intervention on physical activity (PA) levels in preschoolers. Participants were recruited from the 3–5-year-old classes at two preschools. A total of six classrooms and 37 children participated in three conditions: an active reading book read by a researcher (Act_R) trained in active play techniques, an active reading book read by a preschool classroom teacher (Act_T), and a book about health behavior read by both the researcher and the teacher (Sed_H). The order in which classes received each condition was randomized. The Actigraph accelerometer assessed PA. Motor skills were assessed with the Peabody Motor Development Scale, 2nd Edition. Participants spent significantly more time in sedentary behavior during the Sed_H condition compared to Act_R (*p* < 0.000) and Act_T (*p* < 0.008). Participants spent significantly more time in MVPA during Act_R compared to Act_T (*p* = 0.030), Act_T compared to Sed_H (*p* < 0.001), and Act_R compared to Sed_H (*p* < 0.001). The amount of MVPA participation within the active reading sessions was not dependent upon the level of fundamental motor skill competence. Active reading books may provide a feasible method to incorporate physical activity and active play into the preschool day.

## 1. Introduction

Current estimates suggest that globally 1.4 billion adults do not meet the recommended amount of physical activity associated with health benefits [1]. Further estimates show that globally almost 500 million new preventable non-communicable diseases will occur between 2020–2030 if there is no change in the current prevalence of physical inactivity [2]. As health behaviors, particularly physical activity participation and sedentary behavior, are developed in early childhood [3,4,5], it is essential that physical activity opportunities are available from an early age.

Current recommendations state that young children should be active for at least fifteen minutes every hour [6]. Further recommendations from the World Health Organization suggest that children aged three to five years need at least 180 min of daily physical activity with at least 60 min of moderate to vigorous physical activity (MVPA) [7]. Unfortunately, the prevailing literature shows that preschool aged children are spending 80% of their day participating in sedentary behaviors [8] and not meeting physical activity recommendations [9]. Thus, several researchers have developed a variety of physical activity interventions aimed at improving physical activity in the school settings [10,11,12]. In general, these interventions have a small-to-moderate effect, with teacher-led interventions being most successful [13]. Notably, Carroll and colleagues [14] found that teacher-demonstrated modeling was significantly related to light physical activity and MVPA. Although preschool teachers recognize that young children need to move [15], the pressure to focus more on “academic time” and less on “physical activity opportunities” [16,17] contributes to inadequate physical activity opportunities in preschool settings [18]. Therefore, teachers may be more responsive to physical activity interventions that are based within academic learning objectives.

Carroll and colleagues noted small group and reading times displayed the lowest levels of light physical activity and MVPA, suggesting that these times might be prudent for physical activity interventions [14]. Reading books to children is considered one of the most influential activities that foster children’s language and literacy development [19,20,21,22]. Furthermore, the National Association for the Education of Young Children, an overarching accreditation agency in the United States, has recognized that reading to children is a developmentally appropriate practice [23]. Considering the importance of reading to children, utilizing active reading books during reading time may be a pathway to increase physical activity. 

An active reading book is designed to include aspects of active play, often having the reader demonstrate a move and asking the children to repeat the move with them. Active play is a form of physical activity, which results in an increase in energy expenditure, use of gross motor skills, and is enjoyable [24]. Furthermore, several organizations have stressed the importance of play in preschool settings [25,26] as play impacts multiple areas of development and we learn and gain skills through play [27,28]. Some examples of active reading books utilized in early childhood education include: From Head to Toe [29], Gallop! [30], and Walker Finds His Wiggle by Sheila Booth-Alberstadt and the University of West Florida (UWF) Wiggle Team [31]. However, the extent of physical activity participation during an active reading activity remains unclear, as well as whether teachers can elicit similar physical activity participation compared to trained researchers. Additionally, as fundamental motor skills are the basis for more advanced physical movements and correlated with physical activity [32,33], it is important to determine if this type of intervention is effective across levels of motor competence. Therefore, the aims of this study were to assess the levels of physical activity during an active reading activity and to determine potential differences in preschoolers’ physical activity participation among three conditions: an active reading book read by research personnel trained in active play techniques (Act_R), an active reading book read by preschool teachers (Act_T), and a preschool book discussing healthy behaviors without physical activity prompts (Sed_H). Additionally, this study examined whether the level of competency in fundamental motor skills influenced the level of physical activity. We hypothesized that the active reading activity with the researcher and the teacher would have significantly higher levels of MVPA compared to the traditional reading book. We also hypothesized that the level of motor proficiency would not impact the amount of physical activity participation.

## 2. Materials and Methods

### 2.1. Participants and Setting

Prior to any data collection, parental consent was obtained, and this study was approved by Auburn University’s Institutional Review Board for Research Involving Human Subjects (#10-217 MR 1009, approved on 9 June 2021) and conformed to the latest Declaration of Helsinki. This study served as a follow-up to a large, randomized control trial. For this study, participants were recruited from three-to-five-year-old classes at two preschool centers. Both centers were operated by the same entity and were paid for service preschool centers. All lead classroom teachers met or exceeded the requirement for preschool teachers in the state of Alabama, which includes at least 18 h of approved early education or child development coursework from an accredited institution. Three teachers exceeded this requirement and had a bachelor’s degree in education or early childhood education. Classrooms were divided by age, with two classes for 4–5-year-olds, two classes for 3–4-year-olds and two classes for 3-year-olds. A total of 6 classrooms and 37 children across both centers enrolled in the active reading intervention.

These two centers provided childcare from 6:30 a.m. to 6:00 p.m. A typical daily schedule consisted of indoor free play from 6:30 a.m.–8:15 a.m., breakfast from 8:15 a.m.–8:30 a.m., and curriculum instruction time and morning outside time from 8:40 a.m.–12:00 p.m. During this morning period, teachers often implemented a morning move time to help the children transition from breakfast to circle time, where curriculum instruction took place. The afternoon schedule consisted of lunch at 12:00 p.m., naptime 12:30 p.m., afternoon snack at 3:00 p.m., and small groups, centers, and afternoon outside time from 3:15 p.m.–5:00 p.m. Any child who was present after 5:00 p.m. was provided a late afternoon snack and participated in indoor free play and centers until they were picked up.

### 2.2. Procedure

This study was conducted over a three-week period. During the first week of this study, anthropometrics, demographics, and fundamental motor skills were assessed at both centers. In the following two weeks, three experimental reading conditions were implemented at the centers (Table 1) and physical activity was assessed within each condition. As these centers did not have a consistently scheduled read aloud time, the second author met with each teacher prior to the start of the intervention to determine the easiest time for implementation. Five of the classes chose to implement the active reading activities in the transition time between morning move time and circle time. One classroom chose to implement during the transition from afternoon snack to small groups. After discussing times with the teachers, a random number generator randomized the order in which each classroom would receive the intervention conditions. Below is a description of each condition. Table 1 presents the schedule in which the individual classrooms received the conditions.

### 2.3. Experimental Conditions

#### 2.3.1. Active Reading Conditions

The active reading book utilized was: “*Walker Finds His Wiggle*” by Sheila Booth-Alberstadt and the UWF Wiggle Team [31]. The book was developed as part of the “*Let’s Wiggle with 5210*” campaign, whose aim was to improve the overall health of residents in northwest Florida. The campaign targets four key daily behaviors identified by the Florida Department of Public Health: consuming 5 or more fruits and vegetables, limiting recreational screen time to two hours or less, engaging in one or more hours of physical activity, and consuming zero sugary drinks. This book was designed to increase children’s movement while practicing counting as they follow along with several different animal friends. There were two experimental conditions that utilized the active reading book. During the first condition, either the first or second author read the book while verbally prompting and demonstrating the movements to the children (Act_R). The second condition involved one of the classroom teachers reading the book while verbally prompting and demonstrating the movements to the children (Act_T).

#### 2.3.2. Comparative Condition

During the comparative condition, a “sedentary book” or a traditional reading book was read to the children. The “*Play the Walker Wiggle way Featuring Abby Fitt*” book written by Sheila Booth-Alberstadt and the UWF Wiggle Team [34] served as the traditional reading book. This book follows the main character from the active reading book, Walker Wiggle, and his cousin Abby Fitt. Throughout this story, children learn healthy habits including the importance of drinking water, limiting screen time, and participating in active play. The book does not prompt physical activity, nor did the researcher or teacher prompt or demonstrate movements to the children while reading the book. To serve as the best comparison condition, one of the authors (DDW or KES) read half the book, then a classroom teacher read the other half of the book. This condition is referred to as Sed_H.

### 2.4. Measures

#### 2.4.1. Anthropometrics

Date of birth, sex, and race were identified from school records completed by the parent and/or legal guardian. Weight was recorded to the nearest 0.5 kilogram (kg) using a standard beam balance scale with the subject barefoot or in socks. Body height was recorded to the nearest 0.5 centimeter (cm) with the subject barefoot or in socks using a stadiometer. This information was gathered primarily to calibrate the accelerometers. Body mass index (BMI) was calculated as weight (kg) divided by height (m^2^) and classified participants as underweight, normal, overweight, or obese based on CDC BMI growth charts by age and sex.

#### 2.4.2. Physical Activity (PA)

PA was assessed using an Actigraph GTX3 (Pensacola, FL, USA) accelerometer. Throughout the intervention, research personnel would attach the accelerometer to the participant’s non-dominate wrist every morning. In the afternoons, accelerometers were removed prior to the participant leaving for the day. Logs were kept to document time on and off. These times were used for wear time adjustments. All PA data were downloaded and analyzed in ActiLife 6 (Version 6.13.4) and calibrated based on participants’ characteristics. Butte cut points [35] in 10 s epochs were used to categorize activity into light or moderate/vigorous PA. In order to best represent each individual’s wear time, researchers applied personalized filters for each participant based on their accelerometer time on and off logs. Attendance records were examined to determine the typical day length for each participant. Researchers determined that if the monitor was worn for less than half of a child’s typical attendance time, that day was removed from the final analysis. On average, participants were at school for approximately 8 h. Thus, if a child’s wear time was less than 4 h during the day, researchers excluded that day from analysis. Furthermore, due to participants having different wear times (i.e., came to school late, left early), the percentages of time spent being physically active for the total day, inside time, outside time, and time during each experimental reading condition were computed.

#### 2.4.3. Fundamental Motor Skills

The Peabody Developmental Motor Scales, Second Edition (PDMS-2) was used to assess fundamental motor skills [36]. The PDMS-2 is a norm- and criterion-referenced fine and gross motor skill test designed for children from birth through age 5 years and 11 months. This assessment has a high level of reliability content sampling (0.89–0.96), time sampling (0.89–0.94), and interrater reliability (0.89–0.96) [36]. Furthermore, content validity has been determined to be satisfactory [36]. When administering the PDMS-2, researchers and clinicians can assess fine motor through two sub-tests (grasping and visual–motor integration) and gross motor through four subtests (reflexes, stationary, locomotor, and object manipulation). When assessing motor skills with the PDMS-2, each child is tested separately following a testing procedure for their age. Each child who is administered the PDMS-2 is tested individually based on developmental age-based milestones for five subtests (grasping, visual–motor integration, stationary, locomotor, and object manipulation). Reflexes are only assessed in children under the age of one. In the current study, researchers only administered the age-appropriate gross motor subtests for our sample, including stationary skills (SS), locomotor skills (LS), and object manipulation skills (OMS). Individuals administering the PDMS-2 score each task as either performed the task correctly = 2, performed tasks partially = 1, or did not execute the task correctly = 0. Raw scores for each subscale are the sum of points the participant scored [33]. The raw score ranges for each subscale are 6–60 for stationary skills, 6–178 for locomotion skills, and 6–48 for object manipulation skills. Upon calculating raw scores, test administrators use age-appropriate tables, located in the testing manual [36], to convert the raw score of each subtest to a standardized scores (0–20). The sum total of all the standardized scores is used to determine the gross motor quartile (GMQ), which has a range of 41–164. In the current study, researchers used GMQ to determine if participation in MVPA during the active reading sessions differed by level of motor competence.

#### 2.4.4. Statistical Analysis

All quantitative analyses were performed with SPSS 26.0. An a priori sample size utilizing G power was calculated using a medium (0.25) and large (0.40) effect size, suggesting a sample range between 28 and 12 to achieve 0.80 power with an alpha level of 0.05. A repeated measures ANOVA examined differences in sedentary, light, and moderate/vigorous PA. Experimental reading condition (Act_T, Act_R, and Sed_H) served as the within condition. The significance level was set at *p* < 0.05. Post hoc analysis followed if appropriate. All analyses met the assumption for sphericity (*p* > 0.05). A logistic regression ascertained the effects of the level of fundamental motor skill, represented by the GMQ score from the PDMS, on the likelihood of participation in MVPA during the active reading sessions. A median split for GMQ separated the sample into two groups, representing higher and lower scores on the PMDS.

## 3. Results

### 3.1. Participants

Thirty-seven preschoolers provided parental consent and assent to participate in the study, with 29 attending at least one of the experimental conditions. Nineteen participants attended all three reading sessions and wore the Actigraph monitor for more than half of the day. Table 2 shows demographics for all participants.

### 3.2. Physical Activity

Table 3 shows indoor and outdoor PA for each of the three conditions. Table 4 shows the results for the repeated measures ANOVA.

#### 3.2.1. PA during the Active Reading Conditions

**Figure 1:**
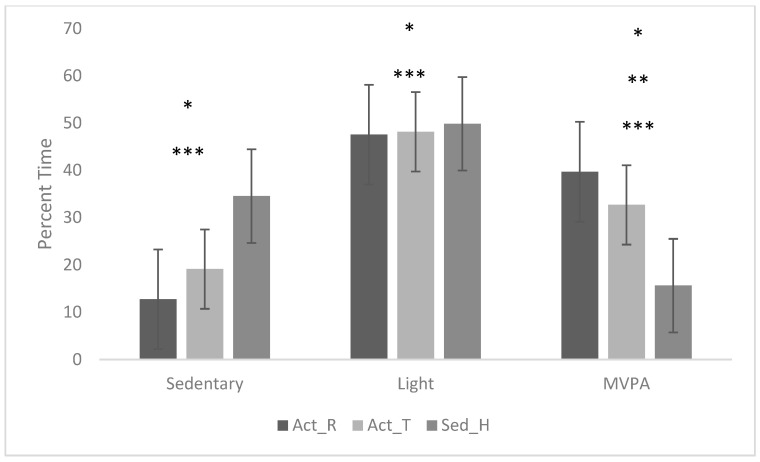
Percentage of time spent in sedentary behavior, light PA, and MVPA during experimental conditions. * = significance between Act_R and Sed_H; ** = significance between Act_R and Act_T; *** = significance between Act_T and Sed_H.

The experimental reading conditions were approximately 10 min long. The repeated measures ANOVA showed a significant effect of time spent in sedentary behavior, light PA, and MVPA during the active reading conditions. Post hoc analysis showed that participants spent significantly more time in sedentary behavior during the Sed_H condition compared to Act_R (*p* < 0.000) and Act_T (*p* < 0.008). Participants also spent significantly more time in light PA during the Sed_H condition compared to Act_R (*p* = 0.003) and Act_T (*p* = 0.035). Participants spent significantly more time in MVPA during Act_R compared to Act_T (*p* = 0.030), Act_T compared to Sed_H (*p* < 0.001) and Act_R compared to Sed_H (*p* < 0.001). Figure 1 shows the differences in PA within the active reading conditions.

#### 3.2.2. Outdoor PA

Outdoor time was approximately 28 min in length. During outdoor time, participants spent approximately 16 min or 50% of their time in MVPA. The results showed that during the Act_T condition, participants spent less time in MVPA compared to the Act_R (*p* = 0.042) and Sed_H (*p* = 0.021) conditions. Participants also spent more time in sedentary behavior during the Act_T condition compared to the Act_R condition (*p* = 0.001). There were no differences in light PA outdoors between the three conditions.

#### 3.2.3. Indoor PA

Participants spent on average 20% in MVPA and 37% in light PA while indoors. For indoor daily PA, there were no significant differences in the amount of time spent in sedentary behavior, light PA, or MVPA between the three conditions.

### 3.3. MVPA and Motor Competence

The logistic regression model examined the relationship between fundamental motor skill competence and participation in MVPA during the active reading sessions. The median score for the PDMS was 100, with 16 cases falling below the median and 16 cases above the median. The model for active reading by the researcher was not statistically significant χ^2^(1) = 3.64, *p* = 0.82. The Nagelkerke R^2^ was 0.00, and the model correctly classified 51.7% of cases. The model for active reading by the teacher was not statistically significant χ^2^(1) = 8.83, *p* = 0.35. The Nagelkerke R^2^ was 0.00, and the model correctly classified 54.2% of cases. These results indicate that the amount of MVPA participation within the active reading sessions was not dependent upon the level of fundamental motor skill competence.

## 4. Discussion

This study examined if an active reading book elicited physical activity in preschoolers during an acute active reading intervention. Our results show that the utilization of an active reading book resulted in significantly higher amounts of MVPA compared to a traditional read aloud activity. These results partially support our hypothesis, as the active reading activity did elicit MVPA; however, the trained researcher experimental condition resulted in significantly higher amounts of MVPA compared to the preschool classroom teacher. As hypothesized, the amount of MVPA accrued during the active reading activity was not impacted by fundamental motor skill competency.

It is well established that the preschool environment and teachers’ practices in particular impact daily physical activity. Where teachers provide physical activity prompts or model physical activity behavior [14], children are more consistently active [37,38]. However, educators may be reluctant to provide physical activity opportunities, due to their own low levels of self-efficacy [37,39], lack of training [40] or lack of resources [18]. Teachers have expressed discomfort and a lack of confidence in providing physical activity opportunities, and consequently tend to avoid incorporating them [41]. Furthermore, teachers have expressed that academic learning and school readiness are a higher priority than movement activities [17]. This study employed an acute read aloud activity to elicit physical activity. The results showed that children spent approximately 39% percent of the time in MVPA when the active reading book was read by the researcher and 27% when read by the teacher, compared to only 10% during the comparative condition. The active reading condition resulted in approximately 7 min of PA, with a little over three minutes of MVPA. Webster and colleagues reported that a 10 min activity break led by preschool classroom teachers resulted in approximately 3 min and 30% of the time in MVPA, which is slightly lower than our results [42,43]. The teachers within this study were asked to model and demonstrate the activities within the active reading book but given no other training or instructions. Based on our limited results, utilizing active reading books within read aloud segments is a feasible and simple solution for teachers to provide physical activity opportunities. Furthermore, this physical activity opportunity occurred indoors, during times in which MVPA is typically low or not offered [14].

Although children were active within the active reading activities, this did not translate to more physical activity indoors. Teachers stated that the active reading activity replaced their daily indoor movement period. Interestingly, outdoor physical activity was reduced when the teachers read the active reading activity, but not during the other two experimental conditions. Anecdotally, teachers appeared to sit more during outside time on days they read the active reading book, thereby prompting and modeling less physical activity. These results may indicate that a teacher’s current level of physical fitness may impact their ability to offer physical activity opportunities. It appears that although teachers were able to implement the activity with little training, intervention effectiveness may improve by providing teachers information on how much movement children should be doing and how teacher behavior impacts children’s PA. There is also some debate as to whether preschoolers compensate or reduce activity in one domain when activity is increased in another domain [42,43]. Within the context of these results, it appears that physical activity compensation in young children within preschool settings may be more attributed to teacher behavior versus a physiological process and should be further explored.

The preschool years are a time of critical fundamental motor skill development, and previous research has indicated that higher fundamental motor skill competency is associated with higher levels of MVPA [32,33]. Our results indicate that the amount of MVPA participation within the active reading sessions was not dependent upon the level of fundamental motor skill competence. Most of the physical activities within the active reading book were based in locomotor skills, which Webster and colleagues showed was associated with the amount of MVPA within classroom-based physical activity breaks [44]. It is important to note that we utilized the PDMS-2 to measure fundamental motor skills with a combined GMQ score, whereas Webster [44] utilized the Test of Gross Motor Development (TGMD) and examined the two subscales of the TGMD. Due to our limited sample size, we utilized a combined GMQ versus a locomotor subscale. Future studies would benefit from examining the impact of fundamental motor competency on intervention outcomes.

Although early learning centers, preschools, and childcare settings have been targeted for physical activity programming extensively over the past decade, the physical activity domain is often neglected [45]. Instead, a push for enhanced academic outcomes in science and mathematics, as well as programs that support the social and emotional needs have been at the forefront of early childhood education with an emphasis on “school readiness” [46,47]. This paradigm shift, driven by changes in policy, curriculum, resources, and funding, focuses more on measurable outcomes and potentially less on holistic child development. Furthermore, under-resourced schools may not provide teacher training for experiential activities and may have higher teacher turnover [47]. As most physical activity programs utilize teacher training, teacher turnover may impact the level of implementation fidelity for physical activity programs and contribute to reduced efficacy [13]. With these factors in mind, active reading books that guide children and teachers through a movement experience may be an easy solution to incorporate indoor physical activity. Furthermore, as preschool teachers have expressed limited efficacy in implementing physical activity and teaching fundamental motor skills [48,49,50], active reading books may be a method to provide instruction within curriculum-based activities. Additionally, books are often used as a method to connect parents to curriculums and could provide a connection for families to experience school related activities while being physically active.

There are several notably limitations to this study. First, our limited sample reduces generalization to other preschool environments. Future studies would benefit from diverse samples from multiple childcare centers. Second, this was an acute study, and we are not able to determine the long-term effectiveness of utilizing this strategy. Future research should examine how active reading books can be incorporated into physical activity programs and interventions over time. In addition, the impact of physical activity within read aloud activities on reading comprehension, attention, and other academic outcomes should be investigated. It would also be helpful to determine teachers’ perceptions about utilizing physical activity within learning contexts and if active reading strategies is a feasible and sustainable solutions for preschool teachers to provide physical activity opportunities.

## 5. Conclusions

The use of active reading books in preschool classrooms appears to be a favorable approach to increasing preschool physical activity levels while providing teachers support to meet academic curriculum needs. With limited training and acute implementation, “*Walker Finds His Wiggle*” improved preschool physical activity levels in teacher and research implemented conditions. Future studies would benefit from longer implementation while examining outcomes on fundamental motor skills and learning objectives.

## Figures and Tables

**Table 1:** Experimental condition schedule.

Center	Classroom	Week 1			Week 2		
		Day 1	Day 2	Day 3	Day 1	Day 2	Day 3
**A**	Class A	Act_R	Sed_H	Act_T			
	Class B	Sed_H	Act_R	Act_R			
	Class C	Sed_H	Act_T	Act_R			
**B**	Class D				Act_T	Sed_H	Act_R
	Class E				Sed_H	Act_R	Act_T
	Class F				Sed_H	Act_T	Act_R

Note: Act_R = active book read by the researcher, Act_T = active book read by the teacher, Sed_H = healthy behavior book read by both the researcher and the teacher.

**Table 2:** Demographics.

Variable	(*n* = 37)
Age (years)	4.16 ± 0.68
Biological Sex	
Male	18 (48.6%)
Female	19 (51.4%)
Height (m.)	1.05 ± 5.37
Weight (kg.)	17.45 ± 2.34
BMI kg/m^2^	15.54 ± 1.11
BMI percentile	50.54 ± 27.90%

Note: BMI = body mass index.

**Table 3:** Daily indoor and outdoor PA.

	Act_R (*n* = 28)		Act_R (*n* = 29)		Sed_H (*n* = 28)	
	Mins	% of Time	Mins	% of Time	Mins	% of Time
Indoor PA						
Sedentary	139.01 ± 42.25	39.82 ± 10.74%	152.75 ± 38.50	43.15 ± 8.62%	150.29 ± 28.15	42.29 ± 8.52%
Light	131.91 ± 36.0	38.40 ± 9.89%	131.86 ± 28.67	37.47 ± 6.39%	132.64 ± 25.07	36.63 ± 6.65%
MVPA	76.91 ± 25.44	21.71 ± 7.21%	73.52 ± 27.57	19.37 ± 6.89%	74.45 ± 29.23	21.07 ± 9.29%
Outdoor PA						
Sedentary	1.31 ± 1.41	4.45 ± 4.61%	1.71 ± 1.27	7.64 ± 5.50%	1.63 ± 1.75	5.73 ± 5.46%
Light	10.23 ± 4.55	37.17 ± 12.9%	10.23 ± 4.04	43.63 ± 13.7%	9.68 ± 4.07	36.96 ± 13.87%
MVPA	17.45 ± 8.04	57.98 ± 15.98%	15.90 ± 7.99	47.26 ± 18.25%	16.89 ± 7.79	56.78 ± 19.68%
Active Reading						
Sedentary	1.02 ± 0.78	12.73 ± 0.7.49%	1.83 ± 1.73	19.12 ± 19.12%	2.76 ± 1.98	34.54 ± 20.45%
Light	3.81 ± 1.41	47.56 ± 14.21%	4.61 ± 1.96	48.17 ± 16.80%	3.99 ± 1.76	49.84 ± 16.89%
MVPA	3.18 ± 1.58	39.70 ± 15.36%	3.13 ± 2.47	32.70 ± 18.63%	1.25 ± 2.13	15.62 ± 9.86%

**Table 4:** RMANOVA results.

	F	*p*	Eta2	Power
Indoor PA				
Sedentary	0.85	0.436	0.05	0.18
Light	0.38	0.690	0.02	0.11
MVPA	2.18	0.127	0.19	0.42
Outdoor PA				
Sedentary	5.64	0.008	0.26	0.83
Light	2.93	0.071	0.18	0.52
MVPA	4.18	0.028	0.26	0.68
Active Reading				
Sedentary	10.67	<0.001	0.37	0.98
Light	6.56	0.004	0.29	0.87
MVPA	23.84	<0.001	0.58	1.00

## Data Availability

The data presented in this study are available on request from Danielle Wadsworth at wadswdd@auburn.edu. The data are not publicly available as participants are minors and could be identified from the data.

## References

[1] Guthold R., Stevens G.A., Riley L.M., Bull F.C. (2018). Worldwide trends in insufficient physical activity from 2001 to 2016: A pooled analysis of 358 population-based surveys with 1.9 million participants. Lancet Glob. Health.

[2] World Health Organization (2022). Global Status Report on Physical Activity 2022: Country Profiles.

[3] Telama R. (2009). Tracking of physical activity from childhood to adulthood: A review. Obes. Facts.

[4] Biddle S.J., Pearson N., Ross G.M., Braithwaite R. (2010). Tracking of sedentary behaviours of young people: A systematic review. Prev. Med..

[5] Van Ekris E., Wijndaele K., Altenburg T.M., Atkin A.J., Twisk J., Andersen L.B., Janz K.F., Froberg K., Northstone K., Page A.S. (2020). Tracking of total sedentary time and sedentary patterns in youth: A pooled analysis using the International Children’s Accelerometry Database (ICAD). Int. J. Behav. Nutr. Phys. Act..

[6] Institute of Medicine (2011). Early Childhood Obesity Prevention Policies.

[7] World Health Organization (2019). Guidelines on Physical Activity, Sedentary Behaviour and Sleep for Children under 5 Years of Age.

[8] Brown W.H., Pfeiffer K.A., McIver K.L., Dowda M., Addy C.L., Pate R.R. (2009). Social and environmental factors associated with preschoolers’ nonsedentary physical activity. Child. Dev..

[9] Lahuerta-Contell S., Molina-García J., Queralt A., Martínez-Bello V.E. (2021). The role of preschool hours in achieving physical activity recommendations for preschoolers. Children.

[10] Alhassan S., Nwaokelemeh O., Mendoza A., Shitole S., Puleo E., Pfeiffer K.A., Whitt-Glover M.C. (2016). Feasibility and Effects of Short Activity Breaks for Increasing Preschool-Age Children’s Physical Activity Levels. J. Sch. Health.

[11] Bellows L.L., Davies P.L., Anderson J., Kennedy C. (2013). Effectiveness of a physical activity intervention for Head Start preschoolers: A randomized intervention study. Am. J. Occup. Ther..

[12] Pate R.R., Brown W.H., Pfeiffer K.A., Howie E.K., Saunders R.P., Addy C.L., Dowda M. (2016). An intervention to increase physical activity in children: A randomized controlled trial with 4-year-olds in preschools. Am. J. Prev. Med..

[13] Gordon E.S., Tucker P., Burke S.M., Carron A.V. (2013). Effectiveness of physical activity interventions for preschoolers: A meta-analysis. Res. Q. Exerc. Sport.

[14] Carroll A.V., Spring K.E., Wadsworth D.D. (2022). The Effect of a Teacher-Guided and -Led Indoor Preschool Physical Activity Intervention: A Feasibility Study. Early Child. Educ. J..

[15] Gehris J., Gooze R., Whitaker R. (2015). Teachers’ perceptions about children’s movement and learning in early childhood education programmes. Child Care Health Dev..

[16] Logue M.E., Harvey H. (2009). Preschool teachers’ views of active play. J. Res. Child. Educ..

[17] Spring K. (2023). Impact of an 8-Week Active Play Intervention on Child Developmental Outcomes. Ph.D. Thesis.

[18] Razak L.A., Clinton-McHarg T., Jones J., Yoong S.L., Grady A., Finch M., Seward K., d’Espaignet E.T., Ronto R., Elton B. (2019). Barriers to and facilitators of the implementation of environmental recommendations to encourage physical activity in center-based childcare services: A systematic review. J. Phys. Act. Health.

[19] Bus A.G., van IJzendoorn M.H., Pellegrini A.D. (1995). Joint Book Reading Makes for Success in Learning to Read: A Meta-Analysis on Intergenerational Transmission of Literacy. Rev. Educ. Res..

[20] Dickinson D.K., Tabors P.O. (2001). Beginning Literacy with Language: Young Children Learning at Home and School.

[21] Scarborough H.S., Dobrich W. (1994). On the efficacy of reading to preschoolers. Dev. Rev..

[22] Lonigan C.J., Shanahan T. (2009). Developing Early Literacy: Report of the National Early Literacy Panel. Executive Summary. A Scientific Synthesis of Early Literacy Development and Implications for Intervention.

[23] Copple C., Bredekamp S. (2009). Developmentally Appropriate Practice in Early Childhood Programs Serving Children from Birth through Age 8.

[24] Truelove S., Vanderloo L.M., Tucker P. (2017). Defining and measuring active play among young children: A systematic review. J. Phys. Act. Health.

[25] Yogman M., Garner A., Hutchinson J., Hirsh-Pasek K., Golinkoff R.M., Baum R., Gambon T., Lavin A., Committee on Psychosocial Aspects of Child and Family Health, Council on Communications and Media (2018). The Power of Play: A Pediatric Role in Enhancing Development in Young Children. Pediatrics.

[26] Friedman S., Wright B.L., Masterson M.L., Willer B., Bredekamp S. (2021). Developmentally Appropriate Practice in Early Childhood Programs Serving Children from Birth through Age 8.

[27] Brown S.L., Vaughan C.C. (2009). Play: How it Shapes the Brain, Opens the Imagination, and Invigorates the Soul.

[28] Elkind D. (2007). The Power of Play: Learning What Comes Naturally.

[29] Carle E., Gaffal A. (1997). From Head to Toe.

[30] Seder R.B. (2009). Gallop! A Scanimation Picture Book.

[31] Booth-Alberstadt S., UWF Wiggle Team (2017). Walker Finds His Wiggle.

[32] Cliff D.P., Okely A.D., Smith L.M., McKeen K. (2009). Relationships between fundamental movement skills and objectively measured physical activity in preschool children. Pediatr. Exerc. Sci..

[33] Robinson L.E., Wadsworth D.D., Peoples C.M. (2012). Correlates of school-day physical activity in preschool students. Res. Q. Exerc. Sport.

[34] Booth-Alberstadt S., UWF Wiggle Team (2019). Play the Walker Wiggle Way Featuring Abby Fitt.

[35] Butte N.F., Wong W.W., Lee J.S., Adolph A.L., Puyau M.R., Zakeri I.F. (2014). Prediction of energy expenditure and physical activity in preschoolers. Med. Sci. Sports Exerc..

[36] Folio M., Fewell R. (2000). Peabody Developmental Motor Scales.

[37] Bower J.K., Hales D.P., Tate D.F., Rubin D.A., Benjamin S.E., Ward D.S. (2008). The childcare environment and children’s physical activity. Am. J. Prev. Med..

[38] Ward D., Hales D., Haverly K., Marks J., Benjamin S., Ball S., Trost S. (2008). An instrument to assess the obesogenic environment of child care centers. Am. J. Health Behav..

[39] Copeland K.A., Kendeigh C.A., Saelens B.E., Kalkwarf H.J., Sherman S.N. (2012). Physical activity in child-care centers: Do teachers hold the key to the playground?. Health Educ. Res..

[40] Wadsworth D.D., Johnson J.L., Carroll A.V., Pangelinan M.M., Rudisill M.E., Sassi J. (2020). Intervention Strategies to Elicit MVPA in Preschoolers during Outdoor Play. Int. J. Environ. Res. Public Health.

[41] Jones R.A., Gowers F., Stanley R.M., Okely A.D. (2017). Enhancing the effectiveness of early childhood educators and researchers working together to achieve common aims. Australas. J. Early Child..

[42] Beck F., Engel F.A., Reimers A.K. (2022). Compensation or displacement of physical activity in children and adolescents: A systematic review of empirical studies. Children.

[43] Webster E.K., Wadsworth D.D., Robinson L.E. (2015). Preschoolers’ time on-task and physical activity during a classroom activity break. Pediatr. Exerc. Sci..

[44] Webster E.K., Robinson L.E., Wadsworth D.D. (2020). Factors that influence participation in classroom-based physical activity breaks in head start preschoolers. J. Phys. Act. Health.

[45] Jones R.A., Sousa-Sá E., Peden M., Okely A.D. (2019). Childcare physical activity interventions: A discussion of similarities and differences and trends, issues, and recommendations. Int. J. Environ. Res. Public Health.

[46] High P.C., Committee on Early Childhood, Adoption, and Dependent Care and Council on School Health (2008). School readiness. Pediatrics.

[47] Haslip M.J., Gullo D.F. (2018). The changing landscape of early childhood education: Implications for policy and practice. Early Child. Educ. J..

[48] Kolt G.S., Schofield G.M., McLachlan C., Oliver M., Lucas P., Maddison R., Walters S. (2005). Koringa Hihiko, Active Movement Scoping Exercise and Programme Evaluation: Report to Sport and Recreation New Zealand.

[49] Oliver M., Duncan S., Kuch C., McPhee J., Schofield G. (2012). Prevalence of New Zealand children and adolescents achieving current physical activity and television watching recommendations. J. Phys. Act. Health.

[50] Breslin C.M., Morton J.R., Rudisill M.E. (2008). Implementing a physical activity curriculum into the school day: Helping early childhood teachers meet the challenge. Early Child. Educ. J..

